# Open Fireplaces

**Published:** 1861-02

**Authors:** 


OPEN FIREPLACES.—One of the most important physical elements of cheerfulness in domestic life has been removed, by the banishment of the old-fashioned open fireplaces from our dwellings. The excuse for this has been, up to this time, that wood was too costly; but, the introduction of Andrews & Dixon's patent for burning any kind of coal on a level with the floor, and in fireplaces as large and commodious as those formerly used in parlors for burning wood, inaugurates a new method of house-warming; and when the public gets to understand it fully, it is believed that no man of intelligence who owns the house he lives in, would consent to be without the Low-Down Grate; because, without consuming any more fuel, for the amount of heat given out into the room, than an ordinary grate, the labor and trouble and care which attend the keeping up of a wood-fire on a cold day is got rid of; for when the fire is kindled in the morning, it needs no further attention until mid-day, when more coal is added, which lasts until a late hour in the night, without the use of poker or tongs during the whole time. This is our own office experience, now going on two years, the ashes being conducted into the cellar, thus getting rid of the dust and trouble of removal every morning. A fire in a Low-Down Grate in the now dark and doleful New-York parlor, would be more ornamental, and would more wake up to lifelike and enlightened and genial conversation than the costliest painting, the most beautiful piece of statuary, or the most elegant vase of unseasonable flowers. At the close of a dreary winter's day, when the sullen cold increases with increasing darkness, and the fitful wind rattles the falling sleet against the window-panes or the lattice, a few lumps of Liverpool or cannel coal put on the broad bed of glowing anthracite in our Low-Down Grate, brings every child into our study; and the smaller ones, especially, sit down on the floor around the hearth, and amuse themselves indefinitely, as to time, in watching the clear, dancing flames, while the exclamation often escapes them: "How cozy!" We ourselves know of but one thing better—the pine-knot fire of our younger years in the distant South, with its delightful aroma! But even this is not wanting in some Southern homes; for shipments of the Low-Down Grate have already been made to every State in the South, where the pine-knots can be burned in perfection; for when once lighted on this grate, they will continue to burn until entirely consumed, giving out all that time the luscious piney odor into the room, while the whole of the smoke is rapidly carried off in another direction.

It should be noted that when the ashes are not conveyed away into the cellar, but are received into an ash-box, the grate is elevated some three or four inches, and a poker and blower are needed. The Low-Down Grate is adjusted to the common fireplace, or can be put in the place of the ordinary grate in half a day, at a total expense of thirty or forty dollars, to last for a lifetime.

In reference to this same subject of open fires against stoves and furnaces, Lewis' New Gymnastics says: "In the whole range of possible topics bearing upon human health, *none is more important!* For does it not seriously concern the character of that vital air which we take into our lungs eighteen times every minute? In our climate it is doubtful if any other physiological question is so momentous as this: *How shall we secure pure air within our houses?*

"OPEN FIREPLACES.—In fitting up a house, *an open fire is number one among house blessings!* No other should precede it. If it were at all convenient, it should be a wood-fire, with a large open fireplace. Oh! how it fills the family circle with comfort, satisfaction, and sociability! To keep up the draft, how the entire air of the room is momentarily changed! No matter how full the room may be, the air can never smell close—the carbonic acid, and other excretions of the animal body, can never accumulate! Strange the people will not have this *delightful sun* in their very houses, at any cost or sacrifice! Go without silks, go without broadcloth, go without carpets, go without finery of all kinds, and have this excellent purifier and diffuser of joy in every house. Who would not go miles to visit an old-fashioned log-house, with its great roaring fire? In whose childish reminiscences is not that cracking, rushing fire the noblest and most beautiful of memories?

"And pray, now, why not have it all back again? If a small part of the money we spend in various foolish customs were given to the reïntroduction of this good old-fashioned blessing, how much healthier and happier we all should be!

"OPEN COAL-GRATES.—Next to an open wood-fire, the open coal-grate, with a good draft, is the best means of warming and ventilating. And if, with a good draft, the coal used be bituminous, it is certainly a very excellent fire.

"STOVES AND FURNACES.—If in the shutter of a dark room you open a small aperture, and look in the jet of light as it streams through the room, you will discover that the air is full of floating moats. The air of our houses is always crowded with these. In their ordinary condition, they do not poison the respiratory apparatus and the blood, but it has been proved by some of the first scientific observers that when they are exposed to contact with a heated stove or furnace, they do poison the man. Millions of these particles, which have been thus burnt, come from the stove, and are sent up from the furnace to poison our lungs and blood.

"Make as many holes in the walls as you please, the air is dull, stagnant, and will give you the headache.

"But some one may say, 'The stove has a draft, too.' Yes, that is true, but the amount of air which thus passes out of the room, when compared with the amount of heat emitted, is almost absolutely nothing.

"I am always sorry when I hear the furnace business is prospering. There never ought to be another one put into a dwelling-house.

"The strong tendency to nervous disease which has shown itself in this country, within the last quarter of a century, may in considerable part be charged against stoves and furnaces.

"Most thoughtfully and conscientiously do I believe that consumption would be greatly lessened if these stoves and furnaces were all thrown overboard. And in this I but echo the voice of the wise in my profession."

Although many persons have called at our office, at 42 Irving Place, New-York, to see the operation of our Low-Down Grate, we still give the invitation to "Come and see," any cold day, only do not come later than eight P.M., for at nine we are in bed.

As to the manner in which the Low-Down Grate has been received, the reader may have some idea in the knowledge that almost every educated physician in Philadelphia, where these grates are made, has one or more of them in his house. They have been ordered from Canada, and from almost every State in the Union. One gentleman, from Mississippi, having used one or more of the Low-Down Grates for several years, was so much pleased with their working, in burning wood, and in their adaptation to a Southern climate, that in building one of the finest private mansions in the South he ordered nineteen of these grates, six of which being plated with silver, cost one hundred and forty dollars each. In burning wood-fires in open fireplaces, it is pretty much the business of one person to keep up a couple of fires in cold weather, while our grate needs no attention whatever from the time of kindling in the morning until retiring for the night, except to lay on a little more coal about midday, requiring no poker nor the removal of ashes during the whole day.

